# Outcome of patients with biochemical recurrence of prostate cancer after PSMA PET/CT-directed radiotherapy or surgery without systemic therapy

**DOI:** 10.1186/s40644-023-00543-0

**Published:** 2023-03-17

**Authors:** Sara Harsini, Don Wilson, Heather Saprunoff, Hayley Allan, Martin Gleave, Larry Goldenberg, Kim N. Chi, Charmaine Kim-Sing, Scott Tyldesley, François Bénard

**Affiliations:** 1 BC Cancer Research Institute, Vancouver, BC Canada; 2 Universtity of British Columbia, Vancouver, BC Canada; 3 Vancouver Prostate Centre, Vancouver, BC Canada

**Keywords:** Prostate cancer, PSMA PET/CT, Biochemical recurrence, Radiotherapy

## Abstract

**Background:**

Radiotherapy (RT) and surgery are potential treatment options in patients with biochemical recurrence (BCR) following primary prostate cancer treatment. This study examines the value of prostate-specific membrane antigen (PSMA) positron emission tomography/computed tomography (PET/CT)-informed surgery and RT in patients with BCR treated without systemic therapy.

**Methods:**

This is a post-hoc subgroup analysis of a prospective clinical trial. Inclusion criteria were: histologically proven prostate cancer at initial curative-intent treatment, BCR after primary treatment with curative intent, having five or fewer lesions identified on [^18^F]DCFPyL PET/CT, and treatment with either PET/CT-directed RT or surgery without systemic therapy. The biochemical progression-free survival after PSMA ligand PET/CT-directed RT and surgery was determined. Uni- and multivariate Cox regression analyses were performed for the association of patients’ characteristics, tumor-specific variables, and PSMA PET/CT imaging results with biochemical progression at the last follow-up.

**Results:**

Fifty-eight patients (30 in surgery and 28 in radiotherapy groups) met the inclusion criteria. A total of 87 PSMA-positive lesions were detected: 16 local recurrences (18.4%), 54 regional lymph nodes (62.1%), 6 distant lymph nodes (6,8%), and 11 osseous lesions (12.7%). A total of 85.7% (24 of 28) and 70.0% (21 of 30) of patients showed a ≥ 50% decrease in prostate-specific antigen (PSA) levels after RT and surgery, respectively. At a median follow-up time of 21 months (range, 6–32 months), the median biochemical progression-free survival was 19 months (range, 4 to 23 months) in the radiotherapy group, as compared with 16.5 months (range, 4 to 28 months) in the surgery group. On multivariate Cox regression analysis, the number of PSMA positive lesions (2–5 lesions compared to one lesion), and the anatomic location of the detected lesions (distant metastasis vs. local relapse and pelvic nodal relapse) significantly correlated with biochemical progression at the last follow-up, whereas other clinical, tumor-specific, and imaging parameters did not.

**Conclusions:**

This study suggests that RT or surgery based on [^18^F]DCFPyL PET/CT are associated with high PSA response rates. The number and site of lesions detected on the PSMA PET/CT were predictive of biochemical progression on follow-up. Further studies are needed to assess the impact of targeting these sites on patient relevant outcomes.

**Trial registration:**

Registered September 14, 2016; NCT02899312; https://clinicaltrials.gov/ct2/show/NCT02899312

**Supplementary Information:**

The online version contains supplementary material available at 10.1186/s40644-023-00543-0.

## Background

Prostate cancer is the most common malignancy affecting men worldwide and a major public health issue in developed countries [1]. Approximately, 30–50% of patients who underwent radiotherapy (RT) [2] or 20–40% of patients initially treated with radical prostatectomy [3] may subsequently experience an increase in prostate-specific antigen (PSA) levels, known as biochemical recurrence (BCR), within 10 years [4]. Although rising PSA can predict recurrent disease or metastasis, it cannot localize the recurrence and sensitive imaging modalities with high diagnostic accuracy are required to distinguish among local relapse, oligometastatic disease, and extensive disease and to enable individualized treatment for patients.

It is widely accepted that extensive metastatic disease will need systemic therapies starting with androgen deprivation therapy (ADT); however, there is no uniform guideline regarding the treatment choice and its timing in the setting of either isolated pelvic nodal relapse, or oligometastatic disease (an intermediate state of tumor spread defined as 1–5 lesions in most studies) with potentially more limited metastatic capacity, less aggressive behavior, and amenable to local treatment [5]. The concept of metastasis-directed therapy (MDT), which aims to eradicate limited cancer spread while avoiding or delaying the toxicity associated with the use of systemic therapies, has gained surging interest since its introduction [6]. As prostate cancer metastases may be seeded not only from the primary tumor but also from other metastatic lesions [6], treatment of localized metastases using radiotherapy or surgery may have a role in slowing disease progression, delaying or avoiding systemic therapy, and improving survival [7]. However, disease-specific randomized phase 3 trials comparing MDT with standard of care have yet to be completed [8]. It is still unclear whether early identification and eradication of low-volume recurrences affect the disease course in these patients, and both stereotactic ablative body radiotherapy [9] and surgery can cause complications [10]. Therefore, physicians are challenged with how best to manage patients with oligorecurrent prostate cancer until robust evidence is available to direct treatment strategies.

Despite emerging data supporting local ablative therapies in patients with metastatic castration sensitive prostate cancer, there is a paucity of high-quality evidence to guide treatment decisions following the localization of PSA recurrence using medical imaging. An accurate diagnostic test, capable of identifying all sites of recurrence with high sensitivity is of utmost importance to choose the best therapeutic plan for a single patient. Conventional imaging techniques, widely used in most clinical trials, including bone scintigraphy, pelvic magnetic resonance imaging (MRI) and CT scan, are frequently unable to localize sites of PSA recurrence at an early stage [11]. During the last decade, positron emission tomography/computed tomography (PET/CT) with ^18^F- or ^11^C-labeled choline was considered to be one of the most accurate imaging modalities available for prostate cancer recurrence with varying detection rates based on PSA values and kinetics [12]. Several prostate-specific membrane antigen (PSMA)-based radiopharmaceuticals, including [^68^ Ga]PSMA-11 [13, 14] and [^18^F]PSMA-1007 [15], were then shown to have better detection rates compared to ^11^C- or ^18^F-choline. [^18^F]DCFPyL is a PSMA-targeting PET radiopharmaceutical with high affinity to the PSMA protein with the advantage of a longer half-life (110 min) and easier regional distribution compared to [^68^ Ga]PSMA-11 [16]. We previously reported that a high proportion of patients undergoing [^18^F]DCFPyL PET/CT imaging for biochemical recurrence of prostate cancer had a limited number of lesions, with a single lesion identified in just over 40% of cases [17].

Currently, there is a paucity of data regarding outcome after [^18^F]DCFPyL PET/CT-directed surgical or radiotherapeutic treatment in biochemically recurrent prostate cancer. To address this, we examined the biochemical progression-free survival after [^18^F]DCFPyL PET/CT-based radiotherapy and surgery for patients with oligorecurrent prostate cancer with five or fewer lesions. Given the high and prolonged efficacy of ADT in castration sensitive prostate cancer, patients receiving ADT in addition to MDT will experience significant and prolonged suppression of PSA levels regardless of the efficacy of the image-guided intervention. We elected to study a subgroup of participants who declined to receive ADT and underwent image-guided interventions for the treatment of oligorecurrent prostate cancer identified by PSMA PET/CT imaging. The outcome of the image-guided interventions in these can provide interesting insights into the specificity of PSMA PET/CT imaging and the efficacy of localized treatment approaches in the absence of systemic therapy.

## Methods

### Study design and participants

We present here a post-hoc subgroup analysis of data from an ongoing prospective investigator-initiated clinical trial (clinicaltrials.gov NCT02899312), which enrolled participants with (1) histologically proven prostate cancer with biochemical recurrence after initial curative therapy with radical prostatectomy, with a PSA > 0.4 ng/mL and an additional measurement showing increase; (2) histologically proven prostate cancer with biochemical recurrence after initial curative therapy with RT, with a PSA level > 2 ng/mL above the nadir after therapy; (3) castration resistant prostate cancer with PSA ≥ 2.0 ng/mL with 2 consecutive rises above nadir and castrate levels of testosterone (< 1.7 nm/L); and (4) findings on other imaging modalities examinations that are suspicious for metastatic disease but not conclusive, and excluded individuals if they were (1) medically unstable; (2) unable to lie supine for imaging; (3) unable to provide written consent; (4) ECOG > 2; or (5) unable to fit through the PET/CT bore (70 cm diameter) or exceeded the safe weight of the PET/CT bed (204.5 kg).

Only participants with castration-sensitive disease, meeting inclusion criteria (1) and (2) with five or fewer radiotracer avid lesions on [^18^F]DCFPyL PET/CT (349/633) were included in this study to assess the outcomes for subjects with biochemical recurrence of prostate cancer treated with curative intent (radical prostatectomy, primary radiotherapy, or a combination of both), undergoing either [^18^F]DCFPyL PET/CT-directed radiotherapy or surgery (137/349). Patients who subsequently received any form of systemic treatment prior to/concomitant with the initiation of PET/CT-directed therapy were excluded from the analysis of treatment outcomes (79/137). Fifty-eight patients met the above-mentioned requirements and were included in this analysis. The study has been approved by the UBC/BC Cancer Research Ethics Board and by Health Canada. Written informed consent was obtained from all participants prior to enrollment in the study.

### Procedures

[^18^F]DCFPyL was synthesized and PET/CT scans were performed as previously reported [18, 19]. [^18^F]DCFPyL was synthesized according to a previously published method [18]. After a 4-h fast, participants were injected intravenously with 237–474 MBq [^18^F]DCFPyL (scaled by body weight), which allowed a 10% variation in the target activity. At 120 min following the injection of [^18^F]DCFPyL, patients were imaged from vertex to mid-thigh on a Discovery PET/CT 600 or 690 (GE Healthcare). A non-contrast-enhanced CT scan for localization and attenuation correction (120 kV, automatic mA selection (30–200 mA range) and noise index of 20) was acquired. Immediately after CT scanning, a whole-body PET scan was acquired over 2–4 min/bed position, adjusted for participant girth, and reconstructed with the ordered subset expectation maximization algorithm and point-spread function modeling.

### Image interpretation

Images were interpreted by credentialed nuclear medicine physicians with experience in reporting prostate PET images on Oasis (Segami) or AW Workstation (GE Healthcare). Physicians with access to all clinical data completed a qualitative interpretation case report form recording the number of positive lesions along with their anatomic site (local recurrence, regional nodes, distant nodes, bone, liver, lung, other). After visual qualitative identification of the tumoral lesions, quantitative data was extracted on PET/CT images reconstructed without the time-of-flight option using a MIM workstation (MIM Encore™ version 6.9.4, MIM Software Inc., Cleveland, USA), on the basis of standardized uptake value (SUV) adjusted for the lean body mass (SUL). Maximum SUL (SULmax), metabolic tumor volume (MTV), total lesion glycolysis (TLG), as well as anatomic site of the lesions of each scan were recorded using manually-corrected semi-automatic contours.

### Follow-up and treatment outcomes

This trial did not mandate clinical management based on the results of PSMA PET/CT imaging, which was left to treating physicians and their patients. PSMA PET/CT results were made available to the treating clinician. Subsequent management plans following the PSMA PET/CT were documented for each patient, including the date and type of treatments initiated.

All radiotherapy and surgical procedures undertaken were based on the management decisions of the treating clinician after the PSMA PET/CT, using local institutional protocols. For the purposes of the trial, any site of surgery or targeted radiation treatment was documented. The surgical technique to be used was at the discretion and expertise of the surgeon but had to be in accordance with the best surgical practice available. In the case of radiotherapy, treatment was categorized as prostatic fossa only, pelvic lymph nodes (with or without prostatic fossa), or radiotherapy to distant metastases.

The outcomes recorded were biochemical progression and biochemical progression-free survival. Biochemical progression was defined as a PSA increase ≥ 25% and ≥ 2 ng/mL if PSA was ≥ 2 ng/mL from baseline, or a PSA increase ≥ 25% if PSA was < 2 ng/mL from baseline, and biochemical progression-free survival defined as the time between the start of post-PET radiotherapy/surgery and the time of progression. The proportion of participants achieving a PSA response defined as ≥ 50% decrease from the PSA at the start of treatment occurred at any point after treatment were also reported. Follow-up time was defined as the interval between the date of initiation of radiotherapy/surgery and the last recorded PSA. Patients who did not show PSA progression at the time of the last follow-up were censored at that time point.

### Statistical analysis

Descriptive statistics were used to summarize patient characteristics by treatment group. The log-rank test was used to compare the biochemical progression-free survival for different subgroups, based on PET/CT-directed therapy, number and site of the PSMA-positive lesions. Kaplan–Meier estimates of biochemical progression-free survival were provided for each group. Univariate and multivariate Cox regression analyses were used to evaluate the association of patients’ characteristics, tumor-specific variables and PSMA PET/CT imaging results with biochemical progression-free survival. Cox regression model was tested for proportional hazards assumptions according to Schoenfeld residuals, and no model violated these assumptions. The maximum percentage decline in PSA from the baseline that occurred at any point after treatment was reported for each patient using a waterfall plot. PSA responses of ≥ 50% were compared between different groups using Fisher’s exact test and one-way analysis of variance (ANOVA). Two-sided P values less than 0.05 were considered statistically significant. All analyses were performed with R (version 3.6.0; The R Foundation for Statistical Computing, General Public License).

## Results

Fifty-eight patients (30 in the PET/CT-directed surgery and 28 in the PET/CT-directed radiotherapy groups) diagnosed with oligorecurrent disease on [^18^F]DCFPyL PSMA PET/CT were included in this analysis of outcome after PET/CT-based surgery and radiotherapy for biochemical recurrence following initial curative therapy. The patients were not under ADT at the time of PET/CT imaging even though 26/58 patients had previously received ADT as adjuvant therapy and were deemed castration-sensitive at the time of PET/CT imaging. The patient characteristics are listed in Table 1. The median follow-up time for the whole cohort was 21 months (range, 6–32 months).

**Table 1:** Patient and tumor characteristics

Characteristic	Surgery (*N* = 30)	Radiotherapy (*N* = 28)
Age at diagnosis (years), median (range)	62.5 (49–73)	65 (47–73)
Initial Gleason score		
6	2 (6.67%)	6 (21.43%)
3 + 4	8 (26.67%)	3 (10.71%)
4 + 3	11 (36.67%)	12 (42.86%)
8	4 (13.33%)	1 (3.57%)
9	5 (16.67%)	6 (21.43%)
Primary tumor classification		
p/cT1	0 (0%)	5 (17.86%)
p/cT2	11 (36.67%)	8 (28.57%)
p/cT3	19 (63.33%)	15 (53.57%)
Primary nodal status		
p/cN0	25 (83.33%)	20 (71.43%)
p/cN1	1 (3.33%)	2 (7.14%)
p/cNx	4 (13.33%)	6 (21.43%)
Prior treatment		
RP	7 (23.33%)	7 (25.00%)
RT	8 (26.67%)	11 (39.29%)
RP + RT	15 (50.00%)	10 (35.71%)
Adjuvant ADT prior to PET/CT	16 (53.33%)	10 (35.71%)
D’Amico Score		
High risk	26 (86.67%)	17 (60.71%)
Intermediate risk	4 (13.33%)	6 (21.43%)
Low risk	0 (0%)	5 (17.86%)
Time from diagnosis to PET/CT (years), median (range)	6.5 (1–19)	7.5 (1–17)
Age at PET/CT (years), median (range)	71.5 (62–79)	73.5 (50–81)
PSA at PET/CT (ng/ml), median (range)	2.39 (0.44–17.8)	2.42 (0.46–9.32)
PSA-DT at PET/CT (mo), median (range)	5.6 (1.2–156)	8.4 (0.7–55)
Number of lesions at PET/CT		
1	16 (53.33%)	24 (85.71%)
2	8 (26.67%)	3 (10.71%)
3–5	6 (20.00%)	1 (3.57%)
Site of lesion	***N***^**a**^** = 54**	***N***^**b**^** = 33**
Local recurrence	7 (12.96%)	9 (27.27%)
Regional lymph node	42 (77.78%)	12 (36.36%)
Distant lymph node	3 (5.56%)	3 (9.09%)
Bone	2 (3.70%)	9 (27.27%)
SULmax sum, median (range)	16.41 (2.51–68.95)	6.17 (2.60–74.15)
MTV sum, median (range)	3.38 (1.11–13.89)	2.26 (1.00–29.21)
TLG sum, median (range)	14.16 (1.23–236.12)	7.19 (0.91–281.23)

*RP* radical prostatectomy, *RT* radiation therapy, *ADT* androgen deprivation therapy, *PET/CT* positron emission tomography/computed tomography, *PSA* Prostate specific antigen, *PSA-DT* prostate-specific antigen doubling time, *SULmax* maximum standardized uptake value normalized to lean body mass, *MTV* metabolic tumor volume, *TLG* total lesion glycolysis

^a^Total number of lesions in the PET-directed surgery group

^b^Total number of lesions in the PET-directed radiotherapy group

Eighty-seven lesions (54 in the surgery and 33 in the radiotherapy groups) were defined as positive based on [^18^F]DCFPyL PET/CT results. A total of 52 (96.3%) and 32 lesions (96.9%) were subsequently targeted by PET/CT-informed surgery and radiotherapy, respectively. The lesion types, numbers and uptake characteristics identified on [^18^F]DCFPyL PET/CT are shown in Table 1.

In the surgery subgroup, local relapse was present in 4 of the 30 patients (13.3%). Twenty-four patients (80.0%) had pelvic nodal relapses (with or without local relapse) and two (6.7%) had distant metastases (distant lymph node metastases and/or osseous metastases). A total of 28 (93.3%) patients in this subgroup underwent PET/CT-directed surgery targeting all their PSMA-avid lesions. Two other patients (6.7%) who had both regional lymph node and bone metastases, just underwent salvage lymph node dissection.

In the radiotherapy subgroup, 8 patients (28.6%) were detected with local relapses; 8 patients (28.6%) presented pelvic nodal relapses (with or without local relapse), and 12 (42.8%) with distant metastases (distant lymph node metastases and/or osseous metastases). Among these patients, 8 (28.6%) received local treatment to prostate, 8 (28.6%) received pelvic nodal treatment and 12 (42.8%) underwent radiotherapy for distant metastases. A total of 27 (96.4%) patients in this group underwent PET/CT-directed radiotherapy targeting all their PSMA-avid lesions. However, one patient (3.6%) had both regional lymph node and bone metastases and just received radiotherapy for bone metastasis.

**Fig. 1:**
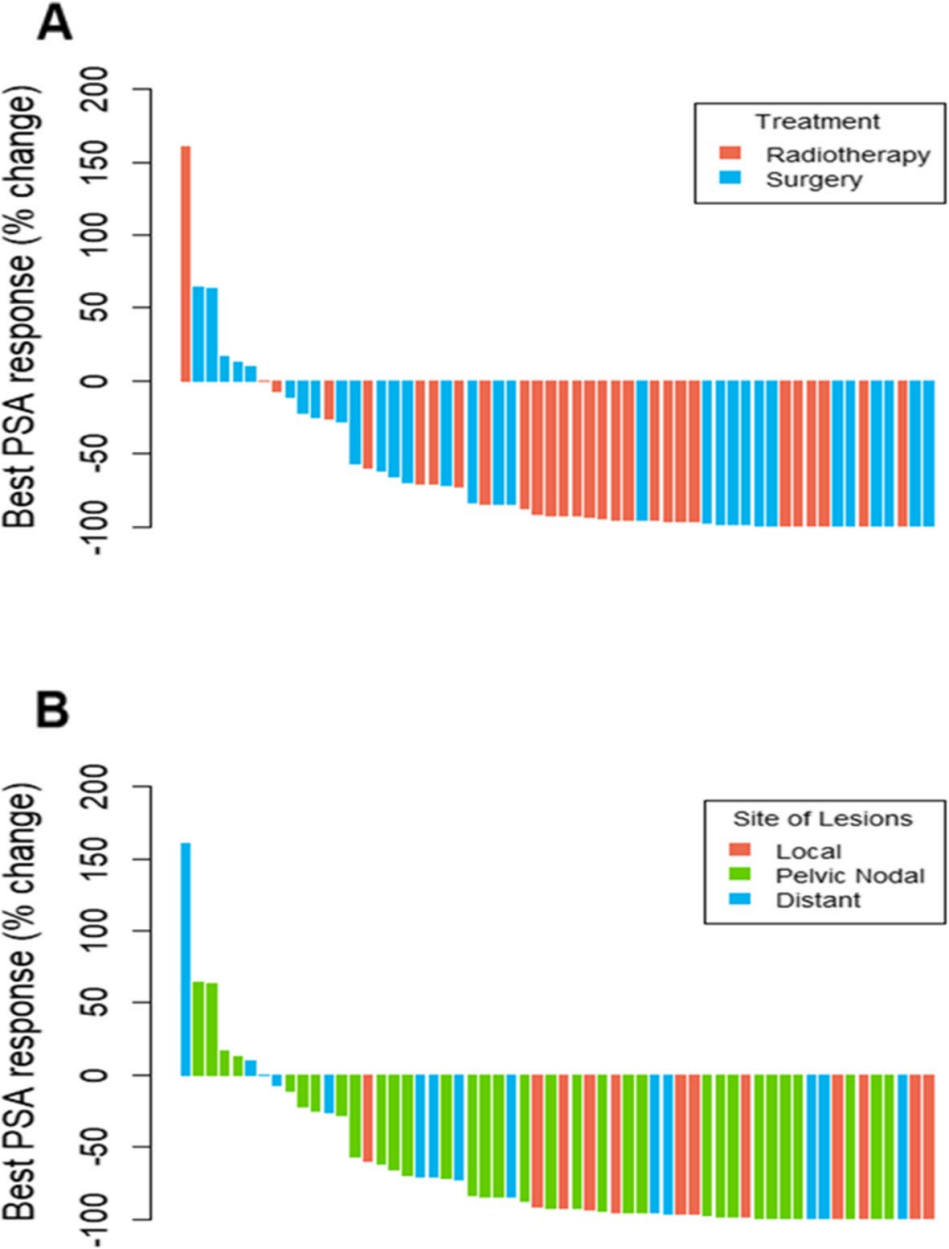
Waterfall plots of the best PSA response after PSMA PET/CT-directed treatment based on the (**A**) PET/CT-directed mode of treatment and (**B**) site of the PSMA-positive lesions

**Fig. 2:**
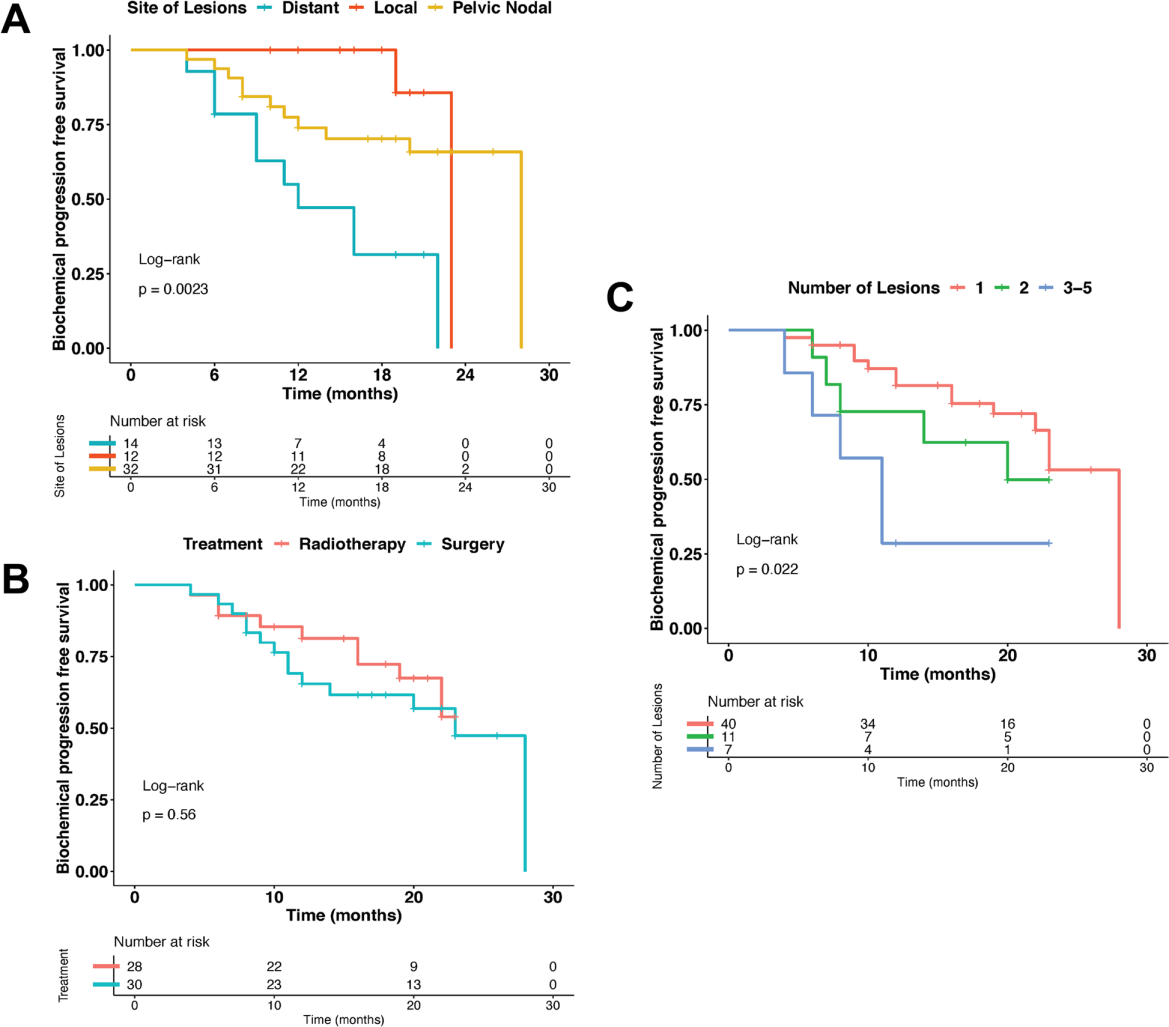
Kaplan–Meier plots comparing the biochemical progression-free survival of patients treated with PSMA PET/CT-directed therapy according to the (**A**) site of lesions, **(B)** PET/CT-directed mode of treatment, and (**C**) number of lesions

The PSA change is depicted as waterfall plots of the largest decline in PSA in Fig. 1. In total, 77.6% of patients who underwent PET/CT-directed therapy had a ≥ 50% PSA response. Survival analysis depicted a median biochemical progression-free survival of 18 months (range, 4 to 28 months) in the study population, with a biochemical progression-free survival rate of 71.7% at one year out of 53 patients followed for at least one year (64.3% in the surgery subgroup and 80.0% in the radiotherapy subgroup). The per-patient analysis of the primary outcome results stratified by the PET/CT-informed mode of treatment (radiotherapy and surgery), number of the PSMA-avid lesions, and their localization (local relapse, pelvic nodal relapse (with or without local relapse), and distant metastasis (distant nodes and/or osseous metastases)), based on which patients received treatment, is reported in Fig. 2, Table 2 and Additional file 1. At the time of the last follow-up, all patients were alive; therefore, the overall survival (OS) was 100%.

**Table 2:** Analysis of the PSA response and the biochemical progression-free survival of patients treated with PSMA PET/CT-informed therapy, broken down by the sites of PSMA-avid relapses

Site	≥ 50% PSA response	One-way ANOVA *P*	Biochemical progression-free survival (mo)^a^	HR (95% CI)	Log-rank *P*
Local relapse^b^	12 (100.00%)	0.07	19.00 (10.00–23.00)	Reference	0.002
Pelvic nodal relapse^c^	24 (75.00%)		19.50 (4.00–28.00)	1.89 (0.41–8.72)	
Distant metastasis^d^	9 (64.29%)		11.50 (4.00–22.00)	6.67 (1.45–30.63)	

*PSA* Prostate-specific antigen; mo, months, *HR* hazard ratio, *CI* confidence interval

^a^Presented as median (range)

^b^Median follow-up time of 19 mo (10–23 mo)

^c^Median follow-up time of 22 mo (8–32 mo)

^d^Median follow-up time of 20 mo (6–22 mo)

On univariate analysis, there was no association between biochemical progression at the last follow-up and the following factors: age at diagnosis; initial T stage and Gleason score; adjuvant ADT prior to PET/CT; age, PSA level and PSA-DT at PET/CT; and SULmax sum, MTV sum and TLG sum of lesions detected on PET/CT. The number of radiotracer-avid lesions detected on PET/CT (3–5 lesions vs. 1 and 2 lesion) and the anatomic location of the detected lesions (distant metastasis vs. local relapse and pelvic nodal relapse) were significantly associated with biochemical progression at the time of last follow-up. On multivariate analysis, a significant association between both the anatomic location of the detected lesions (distant metastasis vs. local relapse and pelvic nodal relapse) and the presence of 2–5 radiotracer-avid lesions on PSMA PET/CT (compared to those with 1 lesion) and biochemical progression at the time of last follow-up was observed (Table 3 and Additional file 2).

**Table 3:** Univariate and multivariate Cox regression analysis for biochemical progression-free survival in patients undergoing PSMA PET/CT-directed radiotherapy or surgery

Characteristics	Univariate analysis			Multivariate analysis		
	**HR**	**95% CI**	***P***^**a**^	**HR**	**95% CI**	***P***^**a**^
Age at diagnosis	0.99	0.93, 1.06	0.80	-	-	-
Initial T stage			0.90			
p/cT1	1.00	Reference		-	-	-
p/cT2	0.75	0.15, 3.77	0.73	-	-	-
p/cT3	0.93	0.21, 4.13	0.92	-	-	-
Initial Gleason Score	1.06	0.69, 1.63	0.77	-	-	-
Adjuvant ADT			0.10			
No	1.00	Reference				
Yes	1.99	0.83, 4.76	0.12	-	-	-
Age at PET/CT	0.97	0.92, 1.03	0.37	-	-	-
PSA at PET/CT	1.06	0.95, 1.18	0.27	-	-	-
PSA-DT at PET/CT	1.01	0.99, 1.03	0.29	-	-	-
Number of lesions			0.02			
1	1.00	Reference		1.00	Reference	
2	1.80	0.63, 5.14	0.27	3.42	1.04, 11.25	0.04
3–5	4.07	1.41, 11.77	0.009	5.04	1.65, 15.42	0.005
Site of lesions			0.002			
Local	1.00	Reference		1.00	Reference	
Pelvic nodal	1.89	0.41, 8.72	0.41	1.13	0.23, 5.48	0.88
Distant	6.67	1.45, 30.63	0.01	6.60	1.39, 31.29	0.02
TLG sum	0.99	0.99, 1.01	0.86	-	-	-
SULmax sum	1.00	0.97, 1.03	0.96	-	-	-
MTV sum	0.97	0.89, 1.06	0.54	-	-	-

*HR* hazard ratio, *CI* confidence interval, *ADT* androgen deprivation therapy, *PSA* prostate-specific antigen, *PSA-DT* prostate-specific antigen doubling time, *TLG* total lesion glycolysis, *SULmax* maximum standardized uptake value normalized to lean body mass, *MTV* metabolic tumor volume

^a^Based on Cox proportional hazards model

## Discussion

The current study is a retrospective analysis of a group of patients who elected to be treated by MDT without systemic therapy. We felt this group was of particular interest to determine the outcome of the PET/CT-directed therapy without the confounding effects of other forms of treatment. For patients who receive ADT in addition to radiotherapy or surgery, it becomes challenging to determine which intervention is responsible for the decline in PSA. Based on a median follow-up of 21 months and an overall number of 58 patients with oligorecurrent disease, our analysis showed a ≥ 50% PSA response in a total of 77.6% of patients after PSMA PET/CT-guided therapy. Patients treated with surgery experienced similar response rates and biochemical progression-free survival as those who underwent radiotherapy. The number and localization of PSMA positive lesions were the major predictive factors for biochemical progression at the time of last follow-up.

Several studies evaluated the benefits and risks of MDT for oligorecurrent/oligometastatic prostate cancer, the majority of which used choline PET/CT as the imaging modality to localize recurrent disease [20–22]. In light of the superior sensitivity of newer PET tracers targeting PSMA to detect smaller lesions, even at low PSA levels [23], choline PET/CT is largely being replaced by PSMA PET/CT to localize oligorecurrent prostate cancer. Deijen et al*.* studied 50 patients with oligometastatic recurrent prostate cancer (49 with PSMA PET, 10 with choline PET) and found that PSMA PET could help select patients with oligometastatic recurrent prostate cancer suitable for SABR at lower PSA levels and subsequently resulted in a significantly longer response duration (34.0 months vs. 14.7 months) and ADT-free survival (32.7 months vs. 14.9 months) compared with choline PET [24]. In the same manner, Mazzola et al*.* observed a higher rate of ADT-free patients following PSMA PET-guided SABR in the setting of oligorecurrent castration-sensitive PC when compared with the choline PET cohort [25]. The suggestion that patients with recurrent prostate cancer may benefit from ablation of their metastatic lesions with RT or by surgical resection mainly derives from studies with fairly heterogeneous patient characteristics [7, 22, 26–28]. Notwithstanding the fact that these studies typically aimed at delaying the initiation of systemic treatments, most of these have used adjuvant ADT to improve oncologic outcomes. This variation in the administration of adjuvant ADT makes the comparison of the progression-free survival (PFS) between studies difficult. It is important to consider differences in study design, patient population, lesions characteristics, and treatment approaches when comparing the results of different studies. Further analysis may be needed to fully understand the impact of these factors, among others, on treatment efficacy and patient outcomes. Studies performed by Herlemann et al*.* [23] and Claeys et al*.* [29] evaluated the benefit of salvage lymph node dissection without ADT in prostate cancer patients with BCR following initial treatment with curative intent and showed median biochemical progression free survival of 12 and 4.1 months, respectively. In another study, Bobrowski et al*.* conducted a retrospective analysis of the outcomes of salvage lymph node dissection in 22 patients with biochemically recurrent prostate cancer and oligometastatic (1–5 lesions) nodal lesions on PSMA PET/CT. After a median follow-up time of 23.1 months, 36.4% of patients achieved a PSA level of < 0.2 ng/mL, with 27.3% maintaining a PSA level of < 0.2 ng/mL, while 40.9% of patients had an initial PSA decline but nadired ≥ 0.2 ng/mL. The median progression-free survival (defined as time to PSA rise, initiation of treatment, or new lesions on imaging) of the cohort was 5.7 months, and the one- and two-year ADT-free survival post-lymph node dissection were 80.6% and 68.2%, respectively [30]. Rogowski et al*.* retrospectively investigated 100 patients with biochemical recurrence/persistence treated with PSMA PET/CT-based salvage elective nodal radiotherapy (with concomitant ADT administration in 83% of cases) and identified one-, 2-, and 3-year biochemical recurrence-free survival of 80.7%, 71.6%, and 65.8%, respectively [31]. In another study of 176 oligometastatic prostate cancer patients treated with PSMA PET/CT-guided MDT delivered with conventional fractionation RT or SABR, Hurmuz et al*.* noted 2-year OS and PFS rates of 87.6% and 63.1%, respectively. Their multivariate analysis suggested an increased number of oligometastases and untreated primary prostate cancer as negative predictors for OS [32]. Koerber et al. [33] assessed the clinical outcome of [^68^ Ga]PSMA-11 PET/CT-guided radiotherapy to selected metastases in 86 patients with recurrent oligometastatic prostate carcinoma, with 61.9% of patients receiving concomitant ADT, and reported a > 50% PSA response in 73.3% of the cohort. After a median follow-up of 26 months, the 3-year OS and PFS were 84% and 55%, respectively. The differences in patient populations between our study and the previous study by Koerber et al*.*, including their baseline characteristics and disease severity, could have influenced the PSA response rates. In the PET-directed radiotherapy group of our study, a lower percentage (60.7%) of patients were high-risk according to the d’Amico risk classification compared to Koerber et al*.*’s study (94.0%). This difference in the risk classification could affect the results as higher-risk patients tend to have a more aggressive form of prostate cancer. Additionally, the number of PSMA-positive lesions detected in the two studies was different. In the PET-directed radiotherapy group of our study, PSMA PET/CT detected 33 lesions in 28 patients (mean 1.17 per patient) compared to Koerber et al*.*’s study where 168 PSMA-positive lesions were detected in 86 patients (mean 1.96 per patient). This difference in the number of lesions detected could also have an impact on the results of the studies. Overall, these differences in the study population should be taken into consideration when interpreting and comparing the results of the two studies.

Prospective studies evaluating the effects of MDT are also in progress. The STOMP trial was the first prospective phase II trial, which randomized 62 patients with a biochemical relapse with three or less extracranial metastases after radical prostate cancer treatment to either MDT (radiotherapy in 25 cases and surgery in 6 cases) or surveillance (31 cases). At a median follow-up time of 3 years, the median ADT-free survival was 21 months in the experimental arm and 13 months in the control arm (*P* = 0.11). No significant correlation was noted between the effect of MDT and the location of metastases (nodal vs. non-nodal) or PSA doubling time (< 3 months vs. > 3 months). Altogether, 74% of patients who received MDT had a PSA decline of any level, compared to just 42% in the surveillance group. The median time until PSA progression was 6 months for the surveillance arm, while it was 10 months for the MDT group (*P* = 0.03) [34]. The ORIOLE trial (observation versus stereotactic ablative radiation for oligometastatic prostate cancer), was a phase II, non-blinded interventional study, which randomized 54 patients with oligometastatic (1–3 metastases) prostate cancer to stereotactic ablative radiation (SABR, 36 cases) or observation (18 cases). After a median follow-up time of 18.8 months, their results showed that 61% of patients in the surveillance group progressed by composite end point (PSA level increase, symptomatic progression, progression detected by conventional imaging, ADT initiation, or death) at 6 months, while only 19% did in the SABR group (*P* = 0.005). The proportion of participants with PSA progression at 6 months was 11% in the SABR group and 50% in the observation arm (*P* = 0.005). Median biochemical progression free survival was not reached for patients treated with SABR, whereas it was 6.4 months for those undergoing observation (*P* = 0.002) [35].

While assessing how MDT affects disease progression, it should be kept in mind that besides the quantity of metastases, which defines the oligometastatic state, qualitative factors, including the microenvironment of the host organs, the aggressiveness of migrating cancer cells, and the time course of metastasis, stand as significant outcome factors warranting further randomized investigations with strict clinical and biological inclusion and exclusion criteria [36, 37]. In order to determine when local therapies alone are oncologically insufficient and should be combined with the systemic therapy, there is a need to precisely distinguish patients with oligometastases from those with subclinical polymetastatic state, and this could be fulfilled through biological cancer staging methods, such as MicroRNA profiling [38, 39].

The present study has some limitations, starting with the fact that this analysis is retrospective in nature, without a randomized control group. The sample size of 58 patients is limited and affects the statistical power. The study mainly included high-risk prostate cancer patients (43 of 58 patients; 74.1%). Hence, caution should be taken when generalizing the observed findings after PSMA PET/CT-guided surgery and RT for patients that had low- or intermediate-risk prostate cancer at the time of initial diagnosis. The lack of comprehensive reporting of toxicities is another limitation of our study. Nevertheless, to our knowledge, there were no reported toxicities necessitating medical intervention or hospitalizations during the follow-up period in our series. Finally, the limited duration of follow-up is another limitation of our study.

## Conclusions

To our knowledge, this is the first study looking at a specific group of patients with biochemical recurrence who underwent [^18^F]DCFPyL PET/CT-guided RT or surgery without the confounding factor of systemic therapy. Without any ongoing antiandrogen therapy, both groups of patients experienced similar PSA response and biochemical progression-free survival. However, the applicability of these results to clinical practice cannot be inferred due to the inherent limitations of the study design, including the small sample size and lack of control group. Further larger, randomized and prospective studies with longer follow-up periods will be necessary to assess the true impact of PSMA PET/CT imaging in the setting of biochemical recurrence of prostate cancer.

## Supplementary Information

**Additional file 1. **Analysis of the PSA response and the biochemical progression-free survival of patients treated with PSMA PET/CT-informed radiotherapy versus surgery, broken down by the sites of PSMA-avid relapses targeted by PET/CT-directed treatment.

**Additional file 2. **Forest plot of the crude HRs based on the site and number of lesions detected on PSMA PET/CT, using a Cox proportional hazards regression model; HR, hazard ratio; CI, confidence interval.

## Data Availability

The data presented in this study are available on request from the corresponding author. The data are not publicly available due to ethical considerations.

## References

[1] Siegel RL, Miller KD, Jemal A (2020). Cancer statistics, 2020. CA Cancer J Clin.

[2] Kestin LL, Vicini FA, Ziaja EL, Stromberg JS, Frazier RC, Martinez AA (1999). Defining biochemical cure for prostate carcinoma patients treated with external beam radiation therapy. Cancer.

[3] Freedland SJ, Humphreys EB, Mangold LA, Eisenberger M, Dorey FJ, Walsh PC (2005). Risk of prostate cancer–specific mortality following biochemical recurrence after radical prostatectomy. JAMA.

[4] Paller CJ, Antonarakis ES (2013). Management of biochemically recurrent prostate cancer after local therapy: evolving standards of care and new directions. Clin Adv Hematol Oncol.

[5] Tree AC, Khoo VS, Eeles RA, Ahmed M, Dearnaley DP, Hawkins MA (2013). Stereotactic body radiotherapy for oligometastases. Lancet Oncol.

[6] Gundem G, Van Loo P, Kremeyer B, Alexandrov LB, Tubio JM, Papaemmanuil E (2015). The evolutionary history of lethal metastatic prostate cancer. Nature.

[7] Ost P, Bossi A, Decaestecker K, De Meerleer G, Giannarini G, Karnes RJ (2015). Metastasis-directed therapy of regional and distant recurrences after curative treatment of prostate cancer: a systematic review of the literature. Eur Urol.

[8] Milano MT, Chowdhry AK, Salama JK, Chmura SJ (2019). Signals from SABR-COMET time to move on to phase III studies. Ann Transl Med.

[9] Palma DA, Olson R, Harrow S, Gaede S, Louie AV, Haasbeek C (2019). Stereotactic ablative radiotherapy versus standard of care palliative treatment in patients with oligometastatic cancers (SABR-COMET): a randomised, phase 2, open-label trial. Lancet.

[10] Abdollah F, Briganti A, Montorsi F, Stenzl A, Stief C, Tombal B (2015). Contemporary role of salvage lymphadenectomy in patients with recurrence following radical prostatectomy. Eur Urol.

[11] Cornford P, Bellmunt J, Bolla M, Briers E, De Santis M, Gross T (2017). EAU-ESTRO-SIOG guidelines on prostate cancer. Part II: treatment of relapsing, metastatic, and castration-resistant prostate cancer. Eur Urol.

[12] Castellucci P, Fuccio C, Nanni C, Santi I, Rizzello A, Lodi F (2009). Influence of trigger PSA and PSA kinetics on 11C-choline PET/CT detection rate in patients with biochemical relapse after radical prostatectomy. J Nucl Med.

[13] Park SY, Zacharias C, Harrison C, Fan RE, Kunder C, Hatami N (2018). Gallium 68 PSMA-11 PET/MR imaging in patients with intermediate-or high-risk prostate cancer. Radiology.

[14] Fendler WP, Calais J, Eiber M, Flavell RR, Mishoe A, Feng FY (2019). Assessment of 68Ga-PSMA-11 PET accuracy in localizing recurrent prostate cancer: a prospective single-arm clinical trial. JAMA Oncol.

[15] Giesel FL, Knorr K, Spohn F, Will L, Maurer T, Flechsig P (2019). Detection efficacy of 18F-PSMA-1007 PET/CT in 251 patients with biochemical recurrence of prostate cancer after radical prostatectomy. J Nucl Med.

[16] Pan K-H, Wang J-F, Wang C-Y, Nikzad AA, Kong FQ, Jian L (2021). Evaluation of 18F-DCFPyL PSMA PET/CT for prostate cancer: a meta-analysis. Front Oncol.

[17] Rousseau E, Wilson D, Lacroix-Poisson F, Krauze A, Chi K, Gleave M (2019). A prospective study on 18F-DCFPyL PSMA PET/CT imaging in biochemical recurrence of prostate cancer. J Nucl Med.

[18] Bouvet V, Wuest M, Jans H-S, Janzen N, Genady AR, Valliant JF (2016). Automated synthesis of [18 F] DCFPyL via direct radiofluorination and validation in preclinical prostate cancer models. EJNMMI Res.

[19] Harsini S, Wilson D, Bénard FJD. PSA-Stratified Performance of [18F] DCFPyL PET/CT in Biochemically Recurrent Prostate Cancer Patients under Androgen Deprivation Therapy. Diagnostics. 2022;12(9):2212. 10.3390/diagnostics12092212 PMC9498260 36140613

[20] Boeri L, Sharma V, Nehra A, Kwon E, Karnes RJ (2020). The role of salvage lymph node dissection in nonmetastatic castration-resistant prostate cancer: a single center experience. Urol Oncol.

[21] Ploussard G, Gandaglia G, Borgmann H, de Visschere P, Heidegger I, Kretschmer A (2019). Salvage lymph node dissection for nodal recurrent prostate cancer: a systematic review. Eur Urol.

[22] De Bleser E, Tran PT, Ost P (2017). Radiotherapy as metastasis-directed therapy for oligometastatic prostate cancer. Curr Opin Urol.

[23] Herlemann A, Kretschmer A, Buchner A, Karl A, Tritschler S, El-Malazi L (2017). Salvage lymph node dissection after 68Ga-PSMA or 18F-FEC PET/CT for nodal recurrence in prostate cancer patients. Oncotarget.

[24] Deijen CL, Vrijenhoek GL, Schaake EE, Vogel WV, Moonen LM, Pos FJ (2021). PSMA-11-PET/CT versus choline-PET/CT to guide stereotactic ablative radiotherapy for androgen deprivation therapy deferral in patients with oligometastatic prostate cancer. Clin Transl Radiat Oncol.

[25] Mazzola R, Francolini G, Triggiani L, Napoli G, Cuccia F, Nicosia L (2021). Metastasis-directed therapy (SBRT) guided by PET-CT 18F-CHOLINE versus PET-CT 68Ga-PSMA in castration-sensitive oligorecurrent prostate cancer: a comparative analysis of effectiveness. Clin Genitourin Cancer.

[26] Schmidt-Hegemann N-S, Kroeze S, Henkenberens C, Vogel M, Kirste S, Becker J (2020). Influence of localization of PSMA-positive oligo-metastases on efficacy of metastasis-directed external-beam radiotherapy—a multicenter retrospective study. Eur J Nucl Med Mol Imaging..

[27] Battaglia A, De Meerleer G, Tosco L, Moris L, Van den Broeck T, Devos G (2019). Novel insights into the management of oligometastatic prostate cancer: a comprehensive review. European Urol Oncol.

[28] Slaoui A, Albisinni S, Aoun F, Assenmacher G, Obeid WAH, Diamand R (2019). A systematic review of contemporary management of oligometastatic prostate cancer: fighting a challenge or tilting at windmills?. World J Urol.

[29] Claeys T, Van Praet C, Lumen N, Ost P, Fonteyne V, De Meerleer G (2015). Salvage pelvic lymph node dissection in recurrent prostate cancer: surgical and early oncological outcome. BioMed Res Int.

[30] Bobrowski A, Metser U, Finelli A, Fleshner N, Berlin A, Perlis N (2021). Salvage lymph node dissection for prostate-specific membrane antigen (PSMA) positron emission tomography (PET)-identified oligometastatic disease. Can Urol Assoc J.

[31] Rogowski P, Trapp C, von Bestenbostel R, Eze C, Ganswindt U, Li M (2022). Outcome after PSMA-PET/CT-based salvage radiotherapy for nodal recurrence after radical prostatectomy. Eur J Nucl Med Mol Imaging..

[32] Hurmuz P, Onal C, Ozyigit G, Igdem S, Atalar B, Sayan H (2020). Treatment outcomes of metastasis-directed treatment using 68 Ga-PSMA-PET/CT for oligometastatic or oligorecurrent prostate cancer: Turkish Society for Radiation Oncology group study (TROD 09–002). Strahlenther Onkol.

[33] Koerber SA, Sprute K, Kratochwil C, Winter E, Haefner MF, Katayama S (2021). Clinical outcome of PSMA-guided radiotherapy for patients with oligorecurrent prostate cancer. Eur J Nucl Med Mol Imaging..

[34] Ost P, Reynders D, Decaestecker K, Fonteyne V, Lumen N, DeBruycker A (2018). Surveillance or metastasis-directed therapy for oligometastatic prostate cancer recurrence: a prospective, randomized, multicenter phase II trial. J Clin Oncol..

[35] Phillips R, Shi WY, Deek M, Radwan N, Lim SJ, Antonarakis ES (2020). Outcomes of observation vs stereotactic ablative radiation for oligometastatic prostate cancer: the ORIOLE phase 2 randomized clinical trial. JAMA Oncol..

[36] Kaneda H, Saito Y (2015). Oligometastases: Defined by prognosis and evaluated by cure. Cancer Treatment Communications.

[37] Reyes DK, Pienta KJ (2015). The biology and treatment of oligometastatic cancer. Oncotarget.

[38] Cheng HH, Mitchell PS, Kroh EM, Dowell AE, Chéry L, Siddiqui J, et al. Circulating microRNA profiling identifies a subset of metastatic prostate cancer patients with evidence of cancer-associated hypoxia. PloS one. 2013;8(7):e69239. 10.1371/journal.pone.0069239 PMC3728349 23935962

[39] Lussier YA, Xing HR, Salama JK, Khodarev NN, Huang Y, Zhang Q (2011). MicroRNA expression characterizes oligometastasis (es). PloS one..

