# Supplementary material for: Outcome of patients with biochemical recurrence of prostate cancer after PSMA PET/CT-directed radiotherapy or surgery without systemic therapy
Source: Cancer Imaging. 2023 Mar 17;23:27. doi: 10.1186/s40644-023-00543-0 (PMC10024380; doi:10.1186/s40644-023-00543-0)
Supplement: Supplementary file 1 — Additional file 1. Analysis of the PSA response and the biochemical progression-free survival of patients treated with PSMA PET/CT-informed radiotherapy versus surgery, broken down by the sites of PSMA-avid relapses targeted by PET/CT-directed treatment. [file 40644_2023_543_MOESM1_ESM.docx]

**Additional Table 1.** Analysis of the PSA response and the biochemical progression-free survival of patients treated with PSMA PET/CT-informed radiotherapy versus surgery, broken down by the sites of PSMA-avid relapses targeted by PET/CT-directed treatment.

| **Site** | **Treatment mode** | **≥ 50% PSA response** | **Fisher’s exact *P*** | **Biochemical progression-free survival (mo)^Ψ^** | **HR (95% CI)** | **Log-rank *P*** |
| --- | --- | --- | --- | --- | --- | --- |
| Local relapse^*^ | Surgery (n=4) | 4 (100.00%) | 1.00 | 18.00 (10.00-23.00) | >100 (0-not calculable) | 0.5 |
|  | Radiotherapy (n=8) | 8 (100.00%) |  | 19.00 (12.00-21.00) |  |  |
| Pelvic nodal relapse^†^ | Surgery (n=24) | 16 (66.67%) | 0.08 | 17.50 (4.00-28.00) | <0.01 (0-not calculable) | 0.05 |
|  | Radiotherapy (n=8) | 8 (100.00%) |  | 21.00 (8.00-23.00) |  |  |
| Distant metastasis^‡^ | Surgery (n=2) | 1 (50.00%) | 1.00 | 10.00 (9.00-11.00) | 0.38 (0.07-2.07) | 0.2 |
|  | Radiotherapy (n=12) | 8 (66.67%) |  | 14.00 (4.00-22.00) |  |  |
| Overall^£^ | Surgery (n=30) | 21 (70.00%) | 0.21 | 16.50 (4.00-28.00) | 0.78 (0.33-1.83) | 0.6 |
|  | Radiotherapy (n=28) | 24 (85.71%) |  | 19.00 (4.00-23.00) |  |  |

Ψ Presented as median (range).

* Median follow-up time of 19 mo (10-23 mo).

† Median follow-up time of 22 mo (8-32 mo).

‡ Median follow-up time of 20 mo (6-22 mo).

£ Median follow-up time of 21 mo (6-32 mo).

PSA, Prostate-specific antigen; mo, months; HR, hazard ratio; CI, confidence interval.
