# Supplementary material for: Outcome of patients with biochemical recurrence of prostate cancer after PSMA PET/CT-directed radiotherapy or surgery without systemic therapy
Source: Cancer Imaging. 2023 Mar 17;23:27. doi: 10.1186/s40644-023-00543-0 (PMC10024380; doi:10.1186/s40644-023-00543-0)
Supplement: Supplementary file 2 — Additional file 2. Forest plot of the crude HRs based on the site and number of lesions detected on PSMA PET/CT, using a Cox proportional hazards regression model; HR, hazard ratio; CI, confidence interval. [file 40644_2023_543_MOESM2_ESM.docx]

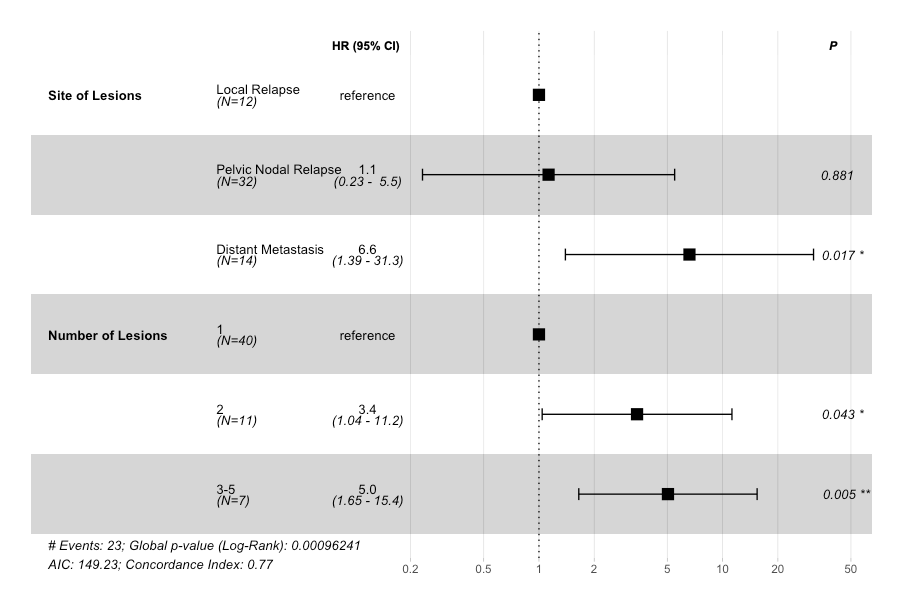


**Additional Figure 1.** Forest plot of the crude HRs based on the site and number of lesions detected on PSMA PET/CT, using a Cox proportional hazards regression model; HR, hazard ratio; CI, confidence interval.
